# Childhood obesity in urban Ghana: evidence from a cross-sectional survey of in-school children aged 5–16 years

**DOI:** 10.1186/s12889-019-7898-3

**Published:** 2019-11-26

**Authors:** John Kuumuori Ganle, Priscilla Pokuaa Boakye, Leonard Baatiema

**Affiliations:** 1 Department of Population,Family and Reproductive Health, School of Public Health, University of Ghana, P.O. Box LG 13, Legon, Accra, Ghana; 2 Stellenbosch Institute for Advanced Study (STIAS), Wallenberg Research Centre at Stellenbosch University, Stellenbosch, 7600 South Africa; 3 Regional Institute for Population Studies, University of Ghana, Accra, Ghana

**Keywords:** Childhood obesity, BMI, In-school children, Lifestyle, Dietary behaviour, Sedentary behaviour, Ghana

## Abstract

**Background:**

Childhood obesity is a growing public health concern in many low-income urban settings; but its determinants are not clear. The purpose of this study is to assess the prevalence of childhood obesity and associated factors among in-school children aged 5–16 years in a Metropolitan district of Ghana.

**Methods:**

A cross-sectional quantitative survey was conducted among a sample of 285 in-school children aged 5–16 years. Pre-tested questionnaires and anthropometric data collection methods were used to collect data. Descriptive, bivariate, binary and multivariate logistic regression statistical techniques were used to analyse data.

**Results:**

Some 46.9% (42.2% for males and 51.7% for females) of the children were overweight. Of this, 21.2% were obese (BMI falls above 95th percentile). Childhood obesity was higher in private school (26.8%) than public school (21.4%), and among girls (27.2%) than boys (19%). Factors that increased obesity risks included being aged 11–16 as against 5–10 years (aOR = 6.07; 95%CI = 1.17–31.45; *p* = 0.025), having a father whose highest education is ‘secondary’ (aOR =2.97; 95% CI = 1.09–8.08; *p* = 0.032), or ‘tertiary’ (aOR = 3.46; 95% CI = 1.27–9.42; *p* = 0.015), and consumption of fizzy drinks most days of the week (aOR = 2.84; 95% CI = 1.24–6.52; *p* = 0.014). Factors that lowered obesity risks included engaging in sport at least 3times per week (aOR = 0.56; 95% CI = 0.33–0.96; *p* = 0.034), and sleeping for more than 8 h per day (aOR = 0.38; 95% CI = 0.19–0.79; *p* = 0.009).

**Conclusion:**

Higher parental (father) educational attainment and frequent consumption of fizzy drinks per week may increase obesity risks among in-school children aged 5–16 years in the Metropolitan district of Ghana. However, regular exercise (playing sport at least 3 times per week) and having 8 or more hours of sleep per day could lower obesity risks in the same population. Age and sex-appropriate community and school-based interventions are needed to promote healthy diet selection and consumption, physical activity and healthy life styles among in-school children.

## Background

Childhood obesity is one of the most important child and public health issues in many parts of the world today [1]. In simple terms, obesity refers to abnormal or excessive fat accumulation resulting from energy imbalance between calories consumed and calories expended [1]. Several factors contribute to obesity. These include sedentary lifestyle, physical inactivity and poor eating habits, including consumption of savoury foods with hidden fats and sugars that impair metabolism [1, 2]. Other factors include biophysiological causes such as genetic causes, insulin resistance, hyperinsulinism, and disruption of the normal satiety feedback mechanisms [1, 2].

Globally, obesity prevalence has nearly tripled since 1970s [1]. For instance, about 1.9 billion adults (18+ years) were overweight in 2016, out of which 650 million were obese [1]. In the same year, 41 million children under age five were overweight or obese [1]. Among children and adolescents aged 5–19, over 340 million were overweight or obese in 2016 [1]. Evidence further suggests that low-income countries harbor majority of obese people [1]. For instance, nearly half of overweight/obese children under-five are in Asia, while one quarter live in Africa [1]. In Africa in particular, the number of overweight or obese children has nearly doubled from 5.4 million to 10.6 million in in 1990 and 2016 respectively [1].

In Ghana, nearly 43% of the adult population is either overweight or obese [3]. Additionally, 45.6% of adult diabetes patients in Ghana are either overweight or obese [3]. Further, recent studies suggest higher overweight/obesity prevalence among women compared to men [4, 5]. For instance, Benkeser, Biritwum and Hill [4] found that 64.9% of Ghanaian women aged 18+ years in the Accra Metropolis were either overweight or obese. Importantly, emerging research suggests high prevalence of childhood obesity in Ghana [6–8]. For example, one study among 5–14 year old children found 17.4% of them to be obese [6].

In current literature, there is recognition of the fact that many obese adults developed the condition in childhood and adolescence [1]. Childhood obesity also has additional consequences, including higher risks of premature death and disability in adulthood, increased risk of fractures, increased future risks of breathing difficulties, heart disease, hepatic impairment, diabetes, insulin resistance, vision problems, cancer, and psychological consequences such as low self-esteem [3, 9, 10]. The difficulty in treating childhood obesity and the social and economic burden of managing the condition are other consequences of childhood obesity [11]. In relation to the economic cost of childhood obesity for instance, one study found a $14.1 billion annual cost for additional prescription drug, emergency room, and outpatient visit healthcare costs annually [12]. Also, an obese 10 year old child who maintains weight gain throughout adulthood has a lifetime medical costs of $19,000 higher compared to a healthy-weight 10-year old who maintains a normal weight throughout life [13]. When compared with a normal weight child, a child who is obese for two consecutive years has a $194 higher outpatient visit expenditure, a $114 higher prescription drug expenditure, and a $12 higher emergency room expenditure [12].

While the evidence on childhood obesity and the potential adverse effects in low-income settings is growing, there is still a general paucity of data on prevalence of childhood obesity in many African settings [14]. Specifically, there is currently limited data on prevalence of obesity in children from 5 to 15 years in many low-income settings [1]. In Ghana for example, a recent systematic view on overweight and obesity epidemic in Ghana highlighted the relative lack of focus on young children and adolescents [3]. In addition, while studies have identified various factors that increase the risk of childhood obesity, including age [15–17], sex [14, 16, 18], educational level of parent/guardian [14, 19], as well as unhealthy diet and physical inactivity [20–24], it is not entirely clear whether these factors are implicated in Ghana’s obesity epidemic. In order to prevent childhood obesity, there is need for context-specific studies to estimate prevalence and identify risk factors [25]. As Guh et al. have argued, one important step in preventing childhood obesity with its associated problems lies in comprehensive studies of its prevalence and associated factors [26]. This study contributes to filling this important knowledge gap by examining prevalence of obesity and associated factors among in-school children in a metropolitan district of Ghana.

## Methods

### Study design

A cross-sectional school-based quantitative survey was conducted among children aged 5–16 in the Tema Metropolitan District of the Greater Accra region of Ghana. The design, implementation and reporting of results followed the Strengthening the Reporting of Observational Studies in Epidemiology (STROBE) checklist for cross-sectional studies.

### Study context

Empirical data collection was done in two of the largest basic schools – one public and the other private - in the Tema Metropolitan district, 30 km East of Accra, the Capital City of Ghana. The metropolis is the second most populous metropolis in the Greater Accra Region. It has a population of 403,934, with nearly everyone living in urban localities [27]. The proportion of children aged 5–16 is estimated to be 29.4% of the total population of the metropolis [27]. There are about 338 basic schools (primary and junior high schools), of which 185 are private and 153 are public schools. The two schools that were purposively chosen for this research were some of the largest in terms of student numbers. The number of students in the private and public schools that were studied were 320 and 610 respectively.

### Study population

We included children from the two selected basic schools who were aged 5-16 years.We however excluded children within the 5-16 year age bracket who had some form of physical disability that could not allow accurate determination of their true height.

### Sample size and sampling

One recent cross-sectional study among 218 in-school children in northern Ghana reported childhood obesity prevalence of 17.4% [6]. Based on this prevalence, and assuming a 95% confidence level, alpha 0.05, 5% worst acceptable margin of error, and power of 80%, we estimated a minimum sample of 221 using Cochran’s sample size estimation formula for cross-sectional studies [28]. We adjusted the minimum sample size upward by 30% to account for non-response and as well increase the power of the study. The final sample size was thus 287.

We used a multistage sampling procedure to select qualified children. In the first stage we obtained registers of all students in each of the two schools. We screened each register to identify children who met our inclusion criteria. In total, 737 children (189 private and 548 public school) from the two basic schools met our inclusion criteria. In stage two, we allocated our total sample of 287 proportionate to the size of the population of children aged 5–16 years in each school. This was to ensure that the sample for each school was commensurate with the size of the population of eligible children. This yielded 191 children from the public school and 94 from the private school. In stage three, we entered the names of all eligible children from each school into excel spreadsheet and gave each a unique number (e.g. 001–548 for the public school, and 001–189 for the private school). The lists of all the numbered children was then exported into a google-based random number generator software, and the required number of children (191 for public school and 94 for private school) for each school was randomly selected. We matched the randomly selected numbers to the corresponding names on our list of eligible children. In stage four, the research team visited the schools to meet all selected children. They were told about the purpose of the study, how they were selected, and what the study procedures involved. All initial questions were addressed in these meetings. As the children were below the age of legal consent (i.e. 18 years), we wrote personalised letters to their parents/guardians. The letters explained the purpose of the study, how the children were selected and what the study procedures were. Information sheets giving further details about the study, including information about ethical approval, rights of their child, informed consent, and contact details of the researchers were included with each letter. Each child delivered the letter to their parent/guardian. Each parent/guardian was given 1 week to decide on their child’s participation in the study. After this period, parents/guardians who consented to their child’s participation were directed to sign or thumbprint the consent form and return it to their child. Only one child did not receive parental consent to participate in the study and was subsequently dropped. All children who received parental consent were required to assent to their parents’ consent. All gave their assent.

### Data collection

The second author (a specialist child health nurse) and two trained research assistants collected the data. Two kinds of data were collected. First, questionnaires were used to collect information on socio-demographic, dietary, and physical activity characteristics of the children. The questionnaire was developed specifically for the purposes of this study based on extensive literature review and expert consultation in Ghana (see Additional file 1). It was however pretested on a total of 30 children aged 5–16 in two other schools not included in the actual study. We tested the reliability of the instrument and found it to be reliable (Cronbach’s alpha coefficient range = 0.80–0.92). The level of reliability observed for both individual items and the entire data collection tool is considered in literature to be good [29]. Details of the test-retest properties are provided in Additional file 2.

Second, anthropometric information such as the weight and height of each child was collected to enable calculation of body mass index (BMI) and obesity status. The anthropometric measurement tools employed were a Weighing Scale (Tanita WB-3000 Digital Doctor Scale, Tanita Corporation of America, Inc., Illinois, USA) to measure the weight of each child in kilograms as they stood on it on a flat hard surface, and a height rod (HR-200 Tanita Wall-Mounted Height Rod, Tanita Corporation of America, Inc., Illinois, USA) to measure the height of children in centimeters.

All data collection was done in the schools during break hours. Children were interviewed one at a time in a specially designated office where maximum confidentiality was assured. Interviews were done in English and three other local dialects (*Ga/Dangme*, *Ewe* and *Twi*) depending on each child’s preference.

### Measurement

The outcome variable of the study is childhood obesity. Similar to previous studies, we used BMI count as a marker of obesity [11]. While adult BMI is easily calculated by dividing body weight in kilograms by height in meters squared, for children and teenagers, BMI requires tuning for age and sex [1]. We used the WHO AnthroPlus software (version 1.0.4) for calculating BMI for children/adolescents aged 5–20 years. BMIs calculated using the WHO AntroPlus were then compared with the CDC curves. Any child with BMI >95th age-sex percentile was considered obese [30]. Our final outcome was dichotomized into ‘obese’ and ‘not obese’.

A number of potential covariates were also measured using the questionnaires, including socio-demographic characteristics like age, sex, educational level of parent/guardian, religion, occupation of parents and obesity history of child’s family; dietary/behavioural factors such as consumption of processed foods and fizzy drinks; and physical activity such as sports, means of transport to school, sleeping hours and playing of computer games other than outdoor games. In particular, sports activity was defined as engaging in any of the following: playing football, basketball, tennis, volley ball and *ampe* (a simple jumping game played by school-aged children - mostly girls - in Ghana and neighbouring countries and usually involving two or more players and requires no equipment), as well as running and cycling. Fizzy drinks were defined as non-alcoholic soft drinks that contain carbonated water, a sweetener (sweetener may be sugar, high-fructose corn syrup, fruit juice, a sugar substitute, or some combination of these), and a natural or artificial flavouring.

### Model specification

In this study, childhood obesity (Y) is the response variable with two binary outcomes: Y = 1, when a child is obese (BMI falls above 95th percentile), and Y = 0, when a child is not obese (BMI falls below 95th percentile). The independent variables for the response variable, Y, are the socio-demographic, dietary and behavioural factors. If Y is the response variable, the probability that a child will be obese is (Y_i_) and the alternative other outcome is (1-Y_i_). Therefore, the odds ratio in favour of a child being obese is

1 $$ OR=\left(\frac{Y_i}{1-{Y}_i}\right)={\beta}_0+{\beta}_i{X}_i $$

Taking the natural log of the odds ratio gives the logit model:

2 $$ \mathit{\ln}\ \left(\frac{Y_i}{1-{Y}_i}\right)={\beta}_0+{\beta}_i{X}_i+{u}_i $$

Where, *‘ln’* is the natural logarithm, *Y*_*i*_ is the probability that a child will be obese, (1 − *Y i)* is the otherwise probability*, β*_0_ denotes the intercept parameter, *X*_*i*_ denotes the explanatory variables, *β*_*i*_ denotes the coefficients to be estimated, and *u*_*i*_ is the error term. From Eq. (2), the model specification for estimating factors that predict obesity status could be represented as follows:

3 $$ In\frac{Yi}{1- Yi}={\beta}_0+{\beta}_1 ag{e}_{child}+{\beta}_2 religio{n}_{child}+{\beta}_3{sex}_{child}+{\beta}_4 ethnicit{y}_{Child}+{\beta}_5 fathe{r}_{educatio{n_{level}}_{child}}+{\beta}_6 mothe{r}_{education\ {level}_{child}}{\beta}_7 numbe{r}_{o{f}_{siblings_{child}}}+{\beta}_8 fathe{r}_{occupatio{n}_{child}}+{\beta}_9 mothe{r}_{ccupatio{n}_{child}}+\dots {\beta}_n+{u}_i $$

Where *In* = natural log; *Y*_*i*_*/(l-Y*_*i*_*)* = odds ratio; *‘ß*_*0*_*’* = intercept term (i.e. probability of a child being obese if all the explanatory variables were equal to zero), *ß*_*1*_ *to ß*_*n*_ = explanatory variables’ coefficients holding all other variables constant, and u_i_ = random error term.

### Data processing and statistical analysis

Following completion of data collection, all questionnaires were first manually examined to check for completeness. Questionnaires were then hand-coded and entered separately into Epi info version 7 by two research assistants. The two data entries were compared, and all data entry errors or inconsistencies were discussed and resolved with the two research assistants who performed the data entry. Following from this, a single database was created, agreed upon, and imported into STATA software for analysis.

Both descriptive and inferential statistical analyses were done. For the descriptive analysis, frequency distributions and proportions were used to summarise categorical variables. Mean and range were computed to summarise continuous variables.

For the inferential statistical analysis, both Chi-square test of independence and fisher’s exact test (for observations with less than 5 cell counts) were performed to first to assess association between childhood obesity and independent variables. This was followed by binary and multivariable logistic regression analysis to estimate odd ratios for factors that showed statistical association at the bivariate level. A 95% confidence level and statistical significance of *p* < 0.05 were assumed in the regression analysis.

## Results

### Characteristics of respondents

A total of 286 children were surveyed; one questionnaire was missing, hence 285 were used for the analysis. Table 1 shows the background characteristics of respondents. Mean age was 11.27(SD = + 4.73), and a little over half (50.5%) were female. Majority (57.5%) were aged 11–16 years. The majority of children reported that their fathers’ (33.0%) and mothers’ (38.7%) highest educational level was basic education. Table 2 also shows essential anthropometric characteristics of respondents. Some 46.9% (42.2 for males and 51.7% for females) of the children were overweight for their age.

**Table 1:** Background characteristics of respondents (*N* = 285)

Characteristic	Private school *n*(%)	Public School *n*(%)	Total *n* (%)
Sex of child			
Male	55 (58.51)	86 (45.0)	141 (49.5)
Female	39 (41.5)	105 (55.0)	144 (50.5)
Age of child (years)			
5–10	51 (54.3)	70 (36.7)	121 (42.5)
11–16	43 (45.7)	121 (56.3)	164 (57.5)
Religion of child			
Christianity	90 (95.7)	177 (92.7)	267 (93.7)
Islam	4 (4.3)	12 (6.3)	16 (5.6)
Traditionalist	0 (0.0)	2 (1.0)	2 (0.7)
Number of siblings			
None	0 (0.0)	1 (0.5)	1 (0.4)
1–4	73 (77.7)	118 (61.8)	191 (67.0)
5–10	18 (19.2)	69 (36.1)	87 (30.5)
11+	3 (3.1)	3 (1.6)	6 (2.1)
Mother’s education			
No education	5 (5.4)	34 (17.8)	39 (13.7)
Basic education	4 (4.3)	106 (55.5)	110 (38.7)
Secondary education	20 (21.5)	35 (18.3)	55 (19.4)
Tertiary education	64 (68.8)	16 (8.4)	80 (28.2)
Father’s education			
No education	5 (5.3)	29 (15.2)	34 (11.9)
Basic education	2 (2.1)	92 (48.2)	94 (33.0)
Secondary education	11 (11.7)	58 (30.4)	69 (24.2)
Tertiary education	76 (80.9)	12 (6.2)	88 (30.9)
Mother’s occupation			
Self-employed	54 (57.5)	176 (92.2)	230 (80.7)
Public sector employee	18 (19.2)	10 (5.2)	28 (9.8)
Private sector employee	22 (23.4)	5 (2.6)	27 (9.5)
Father’s occupation			
Self-employed	164 (85.9)	20 (21.3)	184 (64.6)
Public sector employee	15 (7.8)	23 (24.5)	38 (13.3)
Private sector employee	12 (6.3)	51 (54.3)	63 (22.1)

**Table 2:** Anthropometric characteristics by sex (N = 285)

Characteristic	Male *n*(%)	Female *n*(%)
BMI Percentile		
< 5th	7 (4.9)	5 (3.8)
5th – 85th	75 (52.9)	65 (44.5)
85th – 95th	33 (23.2)	35 (24.5)
> 95th	26 (19.0)	39 (27.2)
Total Weight (kg)	141 (100)	144 (100)
Mean (SD)	34.6 + 10.6	47.8 + 25.0
Height (cm)		
Mean (SD)	135.1 + 19.8	138.3 + 20.7

### Prevalence of childhood obesity

Table 3 shows prevalence of obesity by background of children. Of 46.9% of respondents that were overweight, 21.2% were obese. Some 26.8% of children from the private school were obese compared to 21.4% from the public school. Childhood obesity was also higher among girls (27.2%) than boys (19%).

**Table 3:** Prevalence of obesity by selected socio-demographic characteristics (N = 285)

Characteristic	Not Obese *n*(%)	Obese *n*(%)
Type of School		
Private	69 (73.2)	25 (26.8)
Public	150 (78.6)	41 (21.4)
Sex of child		
Male	114 (81.0)	26 (19.0)
Female	105 (72.8)	39 (27.2)
Age of child (years)		
5–10	105 (86.9)	16 (13.1)
11–16	114 (69.4)	50 (30.6)
Religion		
Christianity	202 (75.8)	65 (24.2)
Islam	14 (85.0)	2 (15.0)
Traditionalist	1 (50.0)	1 (50.0)
Number of siblings		
None	0 (0.0)	1 (100.0)
1–4	146 (76.5)	45 (23.5)
5–10	67 (77.5)	20 (22.5)
11+	5 (86.7)	1 (13.3)
Mother’s education		
No education	36 (91.8)	3 (8.2)
Basic education	80 (72.7)	30 (27.3)
Secondary education	46 (83.6)	9 (16.4)
Tertiary education	56 (70.0)	24 (30.0)
Father’s education		
No education	33 (98.4)	1 (1.6)
Basic education	64 (67.9)	30 (32.1)
Secondary education	60 (86.7)	9 (13.3)
Tertiary education	62 (70.0)	26 (30.0)
Mother’s occupation		
Self-employed	178 (77.4)	52 (22.6)
Public sector employee	21 (73.6)	7 (26.4)
Private sector employee	20 (75.6)	7 (24.4)
Father’s occupation		
Self-employed	145 (78.7)	39 (21.3)
Public sector employee	30 (77.9)	8 (22.1)
Private sector employee	45 (70.8)	18 (29.2)
Overall	225 (78.8)	60 (21.2)

### Factors associated with obesity

To identify factors that significantly predict childhood obesity, chi-square and fisher’s exact tests were first performed between a total of 16 theoretically relevant independent socio-demographic and dietary variables and the outcome variable. The results are shown in Tables 4 and 5. From the bivariate analysis, two socio-demographic factors (age of child [*p* = 0.004], and father’s educational level [*p* = 0.002]), and three dietary/behavioural factors (sports activity per week [*p* = 0.029], regularity of fizzy drinks intake [*p* = 0.004], and sleep hours per day [*p* = 0.001]) were statistically associated with childhood obesity. These factors were pulled into binary and multivariable logistic regression models and odd ratios were estimated. The results are shown in Table 6.

**Table 4:** Socio-demographic factors associated with child obesity (bivariate) (N = 285)

Variable	Not Obese *n*(%)	Obese *n*(%)	*X*^*2*^	*p*-value
Type of School			0.762	0.383
Private	69 (73.2)	25 (26.8)		
Public	150 (78.6)	41 (21.4)		
Sex of child			1.959	0.162
Male	114 (81.0)	26 (19.0)		
Female	105 (72.8)	39 (27.2)		
Age of child (years)			10.783	0.004*
5–10	105 (86.9)	16 (13.1)		
11–16	114 (69.4)	50 (30.6)		
Religion^¥^			2.306	0.270
Christianity	202 (75.8)	65 (24.2)		
Islam	14 (85.0)	2 (15.0)		
Traditionalist	1 (50.0)	1 (50.0)		
Number of siblings^¥^			1.574	0.783
None	0 (0.0)	1 (100.0)		
1–4	146 (76.5)	45 (23.5)		
5–10	67 (77.5)	20 (22.5)		
11+	5 (86.7)	1 (13.3)		
Mother’s education			6.867	0.076
No education	36 (91.8)	3 (8.2)		
Basic education	80 (72.7)	30 (27.3)		
Secondary education	46 (83.6)	9 (16.4)		
Tertiary education	56 (70.0)	24 (30.0)		
Father’s education^¥^			14.537	0.002*
No education	33 (98.4)	1 (1.6)		
Basic education	64 (67.9)	30 (32.1)		
Secondary education	60 (86.7)	9 (13.3)		
Tertiary education	62 (70.0)	26 (30.0)		
Mother’s occupation			0.168	0.919
Self-employed	178 (77.4)	52 (22.6)		
Public sector employee	21 (73.6)	7 (26.4)		
Private sector employee	20 (75.6)	7 (24.4)		
Father’s occupation			1.214	0.545
Self-employed	145 (78.7)	39 (21.3)		
Public sector employee	30 (77.9)	8 (22.1)		
Private sector employee	45 (70.8)	18 (29.2)		

^¥^Fisher’s exact test; *p < 0.05

**Table 5:** Dietary and behavioural factors associated with child obesity (bivariate) (*N* = 285)

Variable	Not Obese	Obese	*X*^*2*^	*p*-value
Sports activity per week			4.7463	0.029*
< 3 days	52 (68.6)	55 (31.4)		
> 3 days	110 (81.8)	68 (18.2)		
Where breakfast is obtained			1.352	0.509
Home	95 (74.3)	80 (25.7)		
Outside of home	52 (71.9)	32 (18.1)		
No breakfast	15 (77.7)	11 (22.3)		
Regularity of eating fruits^¥^			3.139	0.208
Everyday	42 (78.3)	30 (21.7)		
Sometimes	107 (74.6)	89 (25.4)		
Hardly or never	13 (76.5)	4 (23.5)		
Regularity of eating junk foods^¥^			3.042	0.219
Never	8 (92.7)	3 (7.3)		
Monthly	73 (80.8)	47 (19.2)		
Weekly	81 (72.6)	73 (27.4)		
Regularity of fizzy drinks intake			11.150	0.004*
Hardly or never	37 (97.1)	11 (2.9)		
Some days	80 (70.0)	80 (30.0)		
Most days	45 (58.4)	32 (41.6)		
Television exposure			5.009	0.082
Never	10 (86.7)	5 (13.3)		
Some days	53 (68.6)	56 (31.4)		
Everyday	99 (61.5)	62 (38.5)		
Sleep hours per day			14.369	0.001*
Less than 5 h	17 (57.8)	28 (42.2)		
5–8 h	34 (67.9)	37 (32.1)		
8+ h	111 (85.7)	58 (14.3)		

^¥^Fisher’s exact test; *p < 0.05

**Table 6:** Predictors of childhood obesity (multivariate) (*N* = 285)

Variable	cOR (95% C.I.)	*p*-value	aOR (95% C.I.)	*p*-value
Age of child (years)				
5–10 *(ref)*	1		1	
11–16	7.60 (1.29–24.94)	0.032**	6.07 (1.17–21.45)	0.025**
Father’s education				
No education *(ref)*	1		1	
Basic	1.93 (0.73–5.09)	0.185	1.46 (0.51–4.15)	0.483
Secondary	3.86 (1.52–9.78)	0.004**	2.97 (1.09–8.08)	0.032**
Tertiary	4.20 (1.67–10.59)	0.002**	3.46 (1.27–9.42)	0.015**
Sports activity per week^a^				
< 3 days *(ref)*	1		1	
> 3 days	0.58 (0.36–0.95)	0.030**	0.56 (0.33–0.96)	0.034**
Regularity of fizzy drinks intake^b^				
Hardly or never *(ref)*	1		1	
Some days	2.39 (1.06–5.38)	0.035**	2.09 (0.84–5.16)	0.112
Most days	3.36 (1.60–7.06)	0.001**	2.84 (1.24–6.52)	0.014**
Sleep hours per day				
Less than 5 h *(ref)*	1		1	
5–8 h	0.66 (0.31–1.41)	0.286	0.70 (0.31–1.57)	0.391
8+ h	0.32 (0.16–0.63)	0.001**	0.38 (0.19–0.79)	0.009**

^a^Sports activity was defined as engaging in any of the following: playing football, basketball, tennis, volley ball and *ampe* as well as running and cycling)

Ampe is a simple jumping game played by school-age children (mostly girls), in Ghana and neighbouring countries and usually involving two or more players and requires no equipment

^b^Fizzy drinks were defined as non-alcoholic soft drinks that contain carbonated water, a sweetener (sweetener may be sugar, high-fructose corn syrup, fruit juice, a sugar substitute, or some combination of these), and a natural or artificial flavouring

**p < 0.05; *cOR* Crude odds ratio, *aOR* Adjusted odds ratio - aOR for each observation was derived a model which included all variables in the table; *CI* Confidence interval, *ref* Reference category

The results show that children aged 11–16 years had significantly higher odds of being obese as compared to children aged 5–10 years (cOR = 7.60; 95% CI = 1.29–44.9; *p* = 0.032). After adjusting for other variables identified as significant predictors of obesity, this difference was still statistically significant (aOR = 6.07; 95% CI = 1.17–31.45; *p* = 0.025). The risks of childhood obesity generally increased as paternal education increases. Specifically, children whose fathers obtained basic (cOR = 1.93; 95% CI = 0.73–5.09; *p* = 0.185), secondary (cOR = 3.86; 95% CI = 1.52–9.78; *p* = 0.004), and tertiary (cOR = 4.20; 95% CI = 1.67–10.59; *p* = 0.002) education were, respectively, 1.93 times, 3.86 times and 4.20 times more likely to be obese compared to children whose fathers had no education. After adjusting for other variables identified as significant predictors of obesity, the difference between ‘no education’ and ‘secondary education’ (aOR = 2.97; 95% CI = 1.09–8.08; *p* = 0.032), and ‘no education’ and tertiary education (aOR = 3.46; 95% CI = 1.27–9.42; *p* = 0.015) were still statistically significant.

Children who were involved in sporting activities for at least 3 days per week had a 42% significant reduction in their odds of being obese as compared to children who participated in sporting activities for less than 3 days per week (cOR = 0.58; 95% CI = 0.36–0.95; *p* = 0.030). After adjusting for other variables identified as significant predictors of obesity, this association was still statistically significant (aOR = 0.56; 95% CI = 0.33–0.96; *p* = 0.034). Further, children who consume fizzy drinks on some days (cOR = 2.39; 95% CI = 1.06–5.38; *p* = 0.035) and most days (cOR = 3.36; 95% CI = 1.60–7.06; *p* = 0.001), had respectively 2.39 and 3.36 times the odds of being obese as compared to children who hardly or never consume fizzy drinks. These differences were statistically significant. After adjusting for other variables identified as significant predictors of obesity, children who consumed fizzy drinks on most days were still 2.84 times more likely to be obese compared to children who hardly or never consumed fizzy drinks (aOR = 2.84; 95% CI = 1.24–6.52; *p* = 0.014).

Finally, children who slept for more than 8 h per day had 68% reduction in their odds of being obese as compared to children who slept for less than 5 h (cOR = 0.32; 95% CI = 0.16–0.63; p = 0.001). This association was still significant after adjusting for other variables (aOR = 0.38; 95% CI = 0.19–0.79; *p* = 0.009). Furthermore, children who slept between 5 and 8 h, had 34% reduction in their odds of being obese as compared to children who slept for less than 5 h (cOR = 0.66; 95% CI = 0.31–1.41; *p* = 0.286). This association was however not significant.

## Discussion

This study aimed to estimate childhood obesity prevalence and identify key factors among in- school children (5–16 years) in a Metropolitan district of Ghana. Findings highlight childhood obesity as an important public and child health issue that needs attention. To start with, 21.2% of the children in our study were obese, and more children from the private school (26.8%) than the public school (21.4) were obese. Childhood obesity prevalence in our study was higher than the 17.4% prevalence reported in a similar previous study in the northern part of Ghana [6]. However, childhood obesity prevalence in our study is lower than the 43% recently reported for the general adult population in Ghana [6]. This notwithstanding, the relatively high obesity prevalence in our study suggests that children are equally vulnerable to obesity in cities in Ghana. This could be related to the fact that children share the same or similar obesogenic environments with adults, and are therefore increasingly exposed to risk factors such as sedentary life style in urban settings. Also, there is the perception in Ghana about body weight: people are perceived to be living good when they look fat [4]. Consequently, many parents may take steps to ensure that their children conform to this expectation in order to be praised for good parenting. Together with previous studies highlighting growing prevalence of childhood obesity, our findings here suggest a need for promotive health interventions (e.g. healthy eating and physical activity) targeted not only at adults but also children in our study context as well as in other African settings such as Nigeria [15], Uganda [18], and Ethiopia [14] where similarly high levels of childhood obesity have been reported.

Children who were aged 11–16 were 6 times more likely to be obese compared to those aged 5–10 years. It is not entirely clear why this difference exists. However, we believe it could be related to dietary practices as well as sedentary life styles. On diet, children aged 5–10 are more likely to have their dietary choices better regulated than those aged 11–16. For instance, children aged 5–10 are likely to carry home-made meals to school than children aged 11–16 who may have access to money and may therefore purchase their own meals especially in school. This may expose children aged 11–16 to unhealthy diet compared to those aged 5–10, which may affect weight gain and subsequently the BMI of children aged 11–16. As a number of studies have shown, eating outside of home, especially in fast food eateries, is correlated positively with overweight and obesity in children [31]. In terms of sedentary lifestyle, children aged 5–10 may again be more regulated in terms of their sleep time as well as time spend on watching TV and playing computer games within the home environment. Within the school, 5-10 year old children may also be more involved in outdoor games and playground activities than those aged 11–16 who may spend more time doing classroom work. When this is combined with the possibility that children aged 11–16 may be more exposed to unhealthy diet, the likelihood of weight gain and higher BMI could be more in this age group. In line with the recent WHO’s global action plan on physical activity 2018–2030 [2], we recommend targeted interventions such as sport and other physical activities as well as dietary education and nutrition counselling among children aged 11–16 to ensure that they maintain healthy weight.

Further, the risks of childhood obesity appeared to increase with increase in paternal education. This result is very counter-intuitive precisely because better educated parents/guardians generally have greater opportunities for obtaining information related to healthy dietary practices as well as healthy behaviour change information. Therefore, one would expect better outcomes and lifestyle indicators for children whose parents have higher education. This however appears not to be the case in our study. Though counter-intuitive, this result is nevertheless not surprising. This is because in many low-income settings, higher education is often linked to higher socio-economic status, including higher purchasing power. Having higher purchasing power means the ability to afford, for example, personal means of transport such as a car, which may be used to transport children to school compared to children with lowly educated parents who may lack such purchasing power and may walk or ride bicycles. Also, better purchasing power may increase the chances of consumption of more processed foods, unhealthy snacking, consumption of high fat diets within and away from home. This link has been suggested in a number of low-income settings [18]. In addition, better purchasing power may also encourage more sedentary behaviour through access to TV and computer games and related indoor activities that reduce physical activity. Another way that higher education of parents could expose children to obesity risks is work. Parents with higher education are likely to be engaged in paid employment. Tight work schedules may make it difficult for parents to develop healthy meal plans for their households and this could increase consumption of convenient foods which may be unhealthy. Indeed, our results here support growing research evidence that suggests that whereas higher socio-economic status is inversely related to obesity in high-income settings [10], it is positively associated with obesity in low-income settings [32].

Not surprisingly, engaging in sport activities for at least 3 days per week reduced the odds of being obese by 42% compared to children who participated in sport activities for less than 3 days per week. Our results here highlight a need for age- and sex-appropriate sports and physical activity-based interventions such as football, basketball, tennis, volley ball, *ampe* as well as running and cycling among school children. First, while the importance of diet/physical activity in relation to obesity is not new insight, physical activity does represent one side of the energy balance equation, which influences whether energy would be expended leading to a healthy weight or accumulated leading to child obesity. Regular physical activity could potentially lead to reduction in the odds of a child being obese. Therefore, parents and teachers who are the primary caregivers of children should endeavour to increase children’s physical activity. Physical education in schools should be placed on learning time tables for more than three times per week to increase children’s physical activity. Parents should also encourage children to engage in outdoor games. This will however require local government authorities to create save and child-friendly spaces and playgrounds within urban communities – something currently lacking in many urban settings in Ghana - to encourage more outdoor physical activities among children.

Children who consumed fizzy drinks on most days were 2.84 times more likely to be obese compared with those who hardly or never consumed fizzy drinks. This is not surprising given that fizzy drinks typically are sugar-sweetened beverages, and usually have high content of fructose [33]. Also, most sweetened foods are typically calorie dense, which when combined with less physical activity, could easily result in energy imbalance. Indeed, previous studies have found that children who consumed sweetened foods are more likely to be overweight or obese compared to those who do not [3, 8, 19, 22]. In this regard, strategies such as developing healthy meal plans for the household could help regulate children’s consumption of unhealthy foods. Teachers and school authorities could be involved to promote the sale and consumption of more healthy foods especially within the vicinity of educational facilities. In Ghana, most private schools provide lunch at a fee or regulate eating times. For private schools that provide food, they need to develop healthy food timetables for children. In place of fizzy drinks, naturally prepared fruit drinks should be served. Also, parents as much as possible should avoid giving children extra money which they could easily use to purchase fizzy drinks. Parents at home should discourage fizzy drinks consumption by not consuming them. Parents must particularly be examples to their children by consuming healthy and nutritious foods they expect their children to consume as this may drive home the message of healthy eating and living among children. The Government could also help by placing high taxes on fizzy drinks to discourage their consumption.

Also, more sleep hours appeared to reduce the risk of childhood obesity. Children who slept for more than 8 h per day had 62% reduction in their odds of being obese compared to children who slept for less than 5 h. This result support one study among school-aged children in northern Ghana which linked shorter sleep hours to increased obesity risks [6]. In contrast, a study conducted in China revealed that short sleep duration was not associated with obesity [34]. It is not entirely clear why this discrepancy exists and further research is required in different contexts to better explore this issue. However, our results do suggest a need for parents to encourage their children to have sufficient sleep. As a consensus statement of the American Academy of Sleep Medicine recommends, children aged 6–12 need between 9 and 12 h of sleep per 24 h on a regular basis to promote optimal health whereas those aged 13–18 should sleep 8 to 10 h per 24 h on a regular basis to promote optimal health [35].

Our study has some limitations. First, although the anthropometric measurement instrument (e.g. weighing scale) were continuously calibrated and monitored, it is possible that extended period of use could have affected the accuracy of some of the measurement. Related to the above, the use of only BMI as screening tool for obesity has limitations. For instance, concerns have been raised regarding the reliability of weight and height measurement in research contexts [36–38]. Nevertheless, BMI is a valuable population-level indicator used widely in epidemiologic research. Second, there could have been recall bias. This is because respondents were asked about events that occurred several weeks before the interview. Some respondents may also have given socially desirable dietary and behavioural responses such as exercise in order to present themselves as leading active lives when in fact this may not be the case. Third, the study was conducted in only two schools and involved only 285 children in one metropolitan district. Therefore, the limitations of generalizability due to the non-representativeness of the sample are acknowledged. Fourth, our statistical analysis approach was largely data-driven. That is, co-variates were selected based on bivariate significance rather than a pre-specified theoretical view of potential causal structure. Consequently, some variables that may be important after conditioning on other variables may have been missed. This is a limitation of our study. Finally, other factors that were not measured in the study such as parental nutrition knowledge, may nevertheless have had an influence on respondents dietary and behavioural behaviours. These limitations notwithstanding, the results provide useful evidence that could inform large-scale research as well as school-based interventions to reduce the risk of childhood obesity in Ghana.

## Conclusions

This study has provided further insights into the prevalence of, and risk factors, for childhood obesity which have implications for interventions to reduce obesity in Ghana and similar contexts elsewhere. Though predictive socio-demographic factors such as age of child and fathers’ educational attainment may not be amenable to intervention to reduce childhood obesity risks, dietary/behavioural factors observed to influence childhood obesity could be targeted to encourage the maintenance of healthy weight and lifestyle among children. Therefore, age and sex-appropriate community and school-based interventions are needed to promote healthy diet selection and consumption, physical activity and healthy life styles among in-school children. Finally, there is need for large-scale community-based population studies in Ghana to estimate obesity prevalence in different contexts and identify risk factors in different populations. Such information could be vital for national policy and intervention development.

## Supplementary information

**Additional file 1:** Reliability testing of data collection instrument

**Additional file 2:** Study questionnaire

## Data Availability

The datasets used and/or analysed during the current study are available from the corresponding author on reasonable request.

## Abbreviations

BMI: Body mass index
CDC: Centre for Disease control and Prevention
TV: Television
WHO: World Health Organisation
