# Supplementary material for: Childhood obesity in urban Ghana: evidence from a cross-sectional survey of in-school children aged 5–16 years
Source: BMC Public Health. 2019 Nov 26;19:1561. doi: 10.1186/s12889-019-7898-3 (PMC6880588; doi:10.1186/s12889-019-7898-3)
Supplement: Supplementary file 1 — Additional file 1: Reliability testing of data collection instrument [file 12889_2019_7898_MOESM1_ESM.docx]

**Supplementary material 2: Reliability testing of data collection instrument**
