# Supplementary material for: Childhood obesity in urban Ghana: evidence from a cross-sectional survey of in-school children aged 5–16 years
Source: BMC Public Health. 2019 Nov 26;19:1561. doi: 10.1186/s12889-019-7898-3 (PMC6880588; doi:10.1186/s12889-019-7898-3)
Supplement: Supplementary file 2 — Additional file 2: Study questionnaire [file 12889_2019_7898_MOESM2_ESM.docx]

**Supplementary material 1: Study questionnaire**

Dear Participant,

The School of Public Health, University of Ghana, is conducting a research on **“Childhood obesity and its associated factors’’** in private and public schools in your town**.** Your school has been randomly chosen to participate in the study from a list of schools in your town. You have also been randomly chosen to participant in this study. We would like to ask you some questions. The questions usually take about 20-40 minutes. All the answers you give will be confidential and will not be shared with anyone other than members of the study team. You do not have to participate in the survey; but we hope you will agree to answer the questions since your views are important. If we ask you any question you do not want to answer, just let us know and we will go on to the next question or you can stop the interview any time.

In case you need more information about the study, you may contact the persons listed on the information sheet that has already been given to you.

Do you have any questions?

Interview Date: …………………………………

ID No …………………………………………

**A. SOCIO-DEMOGRAPHIC INFROMATION**

1. School name:……….……..

2. Age: ……….years old

3. Sex: M F

4. Religion

A] Christianity B] Islam C] Traditionalist D] Other………………………………

5. Place of Residence (Where you live)…………………………….....................................

6. Indicate number of siblings…………………………….

7. What is the highest level of education that your parents have completed?

Mother ……………………………. Father …………………………………….

8. What is the Occupation of your Parents?

Mother…………………………………. Father……………………………………

**B. ANTHROPOMETIC INFORMATION**

9. How would you describe your weight?

Very underweight Slightly underweight About the right weight

Slightly overweight Very overweight

10. Student weight…………………kg

11. Student height…………………cm

12. BMI………………………………kg/m^2^

**C. DIETRY AND FOOD CONSUMPTION INFORMATION**

13. On school days, where do you usually get your breakfast from?

Home School canteen Shop (outside school)

From a friend I don’t eat breakfast

14. Where do you usually get your lunch?

Home School canteen Shop (outside school) From a friend

I don’t eat lunch Other (Please specify)………………………………………..

15. How many times per week do you eat an evening meal together as a family?

0 days 1 day 2 days 3 days 4 days

5 days 6 days everyday

16. How often do you eat fruits?

Everyday Most days Some days Hardly ever or never

17. How often per month do you eat food from a takeaway (e.g., McDonalds, KFC, pizza, etc.)?

Once a month Once a week 2–3 times a week Most days

18. What is your favorite food?.....................................................................................

19. How often do you eat your favourite food?

Once a week 1–3 times a week 4–7 times a week

20. How often do you take fizzy drinks? Daily Most days Some days Hardly or never

**D. PHYSICAL ACTIVITY INFORMATION**

21. How do you get to school?

A] Walking B] Car or bus C] Bicycle or Motor Bike

22. In the last 5 school days, how many times did you walk to or from school? (Please note, walking from home to school and back on 1 day counts as 2 times; walking to school and taking a car home count as 1 time).

Never 2 times 4 times 6 times 8 times

10 times

23. How long does it take you to walk from home to your school?

Less than 15 minutes 15–30 minutes More than 30 minutes

24. In the last 5 school days, how many days after school did you do sports, in which you were physically active?

0 days 1 day 2 days 3 days 4 days 5 days

25. What kind of sports do you partake

Ampe Football Athletics None

26. Do you have a TV in your house?

Yes No

27. How often do you watch TV

Every day Once a week Twice a week

Less than once a week
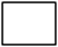
 I don’t watch TV

28. How many hours per day do you spend watching TV?

Less than 1 hour/day 2 hours/day More than 4 hours/day

29. How many hours per day do you spend watching videos or playing games on TV?

Less than 1 hour/day 2 hours/day More than 4 hours/day

30. How much sleep do you usually get each day?

Less than 5 hours Between 5 and 8 hours More than 8 hours
